# Total Glucosides of Paeony Ameliorate Pristane-Induced Lupus Nephritis by Inducing PD-1 ligands^+^ Macrophages *via* Activating IL-4/STAT6/PD-L2 Signaling

**DOI:** 10.3389/fimmu.2021.683249

**Published:** 2021-07-05

**Authors:** Chun-Ling Liang, Hongliang Jiang, Wenxuan Feng, Huazhen Liu, Ling Han, Yuchao Chen, Qunfang Zhang, Fang Zheng, Chuan-Jian Lu, Zhenhua Dai

**Affiliations:** ^1^ Section of Immunology & Joint Immunology Program, Guangdong Provincial Academy of Chinese Medical Sciences, Guangzhou, China; ^2^ The Second Affiliated Hospital of Guangzhou University of Chinese Medicine, Guangzhou, China; ^3^ Gaozhou Hospital of Traditional Chinese Medicine Affiliated to Guangzhou University of Chinese Medicine, Maoming, China; ^4^ Guangdong Provincial Key Laboratory of Clinical Research on Traditional Chinese Medicine Syndrome, Guangzhou, China

**Keywords:** lupus nephritis, total glueosides of paeony (TGP), macrophage polarization, PD-1 ligand, PD-L1, PD-L2

## Abstract

Macrophages, a major subset of innate immune cells, are main infiltrating cells in the kidney in lupus nephritis. Macrophages with different phenotypes exert diverse or even opposite effects on the development of lupus nephritis. Substantial evidence has shown that macrophage M2 polarization is beneficial to individuals with chronic kidney disease. Further, it has been reported that PD-1 ligands (PD-Ls) contribute to M2 polarization of macrophages and their immunosuppressive effects. Total glucosides of paeony (TGP), originally extracted from Radix Paeoniae Alba, has been approved in China to treat some autoimmune diseases. Here, we investigated the potentially therapeutic effects of TGP on lupus nephritis in a pristane-induced murine model and explored the molecular mechanisms regulating macrophage phenotypes. We found that TGP treatment significantly improved renal function by decreasing the urinary protein and serum creatinine, reducing serum anti-ds-DNA level and ameliorating renal immunopathology. TGP increased the frequency of splenic and peritoneal F4/80^+^CD11b^+^CD206^+^ M2-like macrophages with no any significant effect on F4/80^+^CD11b^+^CD86^+^ M1-like macrophages. Immunofluorescence double-stainings of the renal tissue showed that TGP treatment increased the frequency of F4/80^+^Arg1^+^ subset while decreasing the percentage of F4/80^+^iNOS^+^ subset. Importantly, TGP treatment increased the percentage of both F4/80^+^CD11b^+^PD-L1^+^ and F4/80^+^CD11b^+^PD-L2^+^ subsets in spleen and peritoneal lavage fluid as well as the kidney. Furthermore, TGP augmented the expressions of CD206, PD-L2 and phosphorylated STAT6 in IL-4-treated Raw264.7 macrophages *in vitro* while its effects on PD-L2 were abolished by pretreatment of the cells with an inhibitor of STAT6, AS1517499. However, TGP treatment did not affect the expressions of STAT1 and PD-L1 in Raw264.7 macrophages treated with LPS/IFN-γ *in vitro*, indicating a possibly indirect effect of TGP on PD-L1 expression on macrophages *in vivo*. Thus, for the first time, we demonstrated that TGP may be a potent drug to treat lupus nephritis by inducing F4/80^+^CD11b^+^CD206^+^ and F4/80^+^CD11b^+^PD-L2^+^ macrophages through IL-4/STAT6/PD-L2 signaling pathway.

## Introduction

Lupus nephritis (LN) is one of the most common complications in patients with systemic lupus erythematosus and represents a leading cause of end-stage renal failure (1). LN is a complex autoimmune and inflammatory disease resulting from sustained imbalance between pro- and anti-inflammatory immune responses. The substantial infiltration of inflammatory cells in the kidney is a morphologic hallmark of human and experimental lupus nephritis. It is generally accepted that macrophages are the main infiltrating immune cells and play a unique role in the immune-based pathogenesis of lupus nephritis. Infiltrating macrophages can alter the renal microenvironment by interacting with other immune cells, renal stromal cells or propria cells, which then determine the outcome of renal diseases (2, 3). Macrophages with different phenotypes exert diverse or even opposite effects on the development of lupus nephritis (4). A number of studies have demonstrated that inhibiting macrophage infiltration or promoting macrophage M2 polarization is beneficial in the context of chronic kidney disease (5–7). M2 macrophage is capable of inducing Tregs, suppressing the activation of T cells, phagocytosing apoptotic cells and inducing anti-inflammatory factors (8). Further, it has been reported that PD-1 ligands (PD-Ls) contribute to the M2 polarization and immunosuppressive effects of macrophages (9–11). In parallel with effects of PD-Ls on macrophages, adoptive transfer of PD-L2^+^ M2 macrophages delayed the progression of murine experimental autoimmune encephalomyelitis (EAE) (12). In view of the crucial roles for macrophages in the pathogenesis of LN, it is necessary to explore the mechanisms underlying macrophage activation and to seek an efficient therapeutic strategy of treating LN *via* rebalancing macrophage activation.

Total glueosides of paeony (TGPs) are the total glucosides originally extracted from Radix Paeoniae Alba, and TGPs have been approved in China for the treatment of some autoimmune diseases. Indeed, TGP treatment suppressed joint destruction and histological changes in experimental rheumatoid arthritis (13, 14), attenuated skin inflammation in psoriatic patients (15) and animal models (16, 17) and improved renal function in diabetic rats. Mechanistically, TGP treatment suppressed macrophage activation in both gouty arthritis (18) and diabetes in experimental rats (19). In addition, TGP reportedly increased the PD-L1 expression on CD14^+^ monocytes in PBMCs from patients with primary Sjögren’s syndrome (20). Therefore, we hypothesized that TGP could be effective in treating lupus nephritis by inducing PD-Ls^+^ M2 macrophages.

In the present study, we determined whether TGP treatment would suppress lupus nephritis using a pristane-induced murine model of lupus nephritis. We demonstrated that TGP treatment for eight weeks significantly improved renal injury and function in pristane-induced LN mice by inducing PD-L2^+^ macrophages through acting on IL4/STAT6/PD-L2 signaling pathway.

## Materials and Methods

### Mice and Reagents

Female wild-type C57BL/6 mice were obtained from Guangdong Medical Laboratory Animal Center (Fushan, Guangdong, China). All mice were housed in a SPF facility, and all animal experiments were approved by the Animal Ethics Committee of Guangdong Provincial Academy of Chinese Medical Sciences. Pristane and lipopolysaccharide (LPS, L2637, from Escherichia coli O55:B5) was purchased from Sigma-Aldrich (St. Louis, MO, USA). Recombinant mouse IL-4 and IFN-γ were purchased from PeproTech (Rocky Hill, NJ).

Total glucosides of paeony (TGP) were bought from Daosifu Bio-Technique Inc (Nanjing, China). STAT6 inhibitor AS1517499 were obtained from MCE (NJ, USA) and dissolved in dimethylsulfoxide (DMSO). Anti-CD45-BV605 (Clone 30-F11), anti-F4/80-APC (Clone BM8), anti-CD11b-V450 (Clone M1/70), anti-CD206-PE-cy7 (Clone MR6F3), anti-CD86-FITC (Clone GL-1) and anti-CD16/CD32 Abs were bought from BD Biosciences (CA, USA). Anti-PD-L1-Percyp-efluor710 (Clone B7-H1) and anti-PD-L2 (Clone B7-DC) monoclonal antibodies were purchased from eBioscience (San Diego, CA), while rabbit iNOS (Clone D6B6S), Arg1 (Clone D4E3M™), STAT6 (Clone D3H4), p-STAT6 (Clone Tyr641), STAT1 (Clone D1K9Y), p-STAT1 (Clone Tyr701) and PD-L1 Abs (Clone E1J2J™) were purchased from Cell Signaling Technology (Danvers, MA). Mouse F4/80 (Clone C-7) and PD-L2 (Clone TY25) Abs were from Santa Cruz Biotechnology (Santa Cruz, CA). Mouse anti-ds-DNA Ab ELISA kit was purchased from Wuhan Huamei Biotech Co., Ltd (Wuhan, China). Creatinine sarcosine oxidase creatinine assay kits were from Nanjing Jian Cheng Bioengineering Institute (Nanjing, China).

### Animal Treatment

Female C57BL/6 mice (6-8 weeks old) were acclimatized for 2 weeks before experiments and then received a single intraperitoneal injection of 0.5 ml pristane in phosphate-buffered saline (PBS) to induce murine lupus nephritis. About six months (24 weeks) after pristane injection, 24-h urinary protein was measured, and mice with more than 0.4 mg/24 h urinary protein were included in the subsequent experiments. The included mice were randomized into different groups and treated orally without or with TGP (200 mg/kg or 100 mg/kg, prepared with distilled water containing 0.4% CMC-Na) daily for another two months. Serum creatinine (Scr) levels were measured 24 hours after the last drug treatment. Blood, spleen, peritoneal lavage fluids and kidney were also collected 24 hours after the last drug administration.

### Measurement of Renal Function

Proteinuria was measured by collecting 24-h urine every 2 weeks before and during TGP treatment. All mice were prohibited from getting food but allowed for free access to water during the collection of urine samples. The urine samples were centrifuged at 400g for 5 min and tested with Cobas-8000 automatic biochemical analyzer (Roche). Serum creatinine (Scr) and serum anti-dsDNA Ab levels were detected using sarcosine oxidase creatinine assay kits and murine anti-dsDNA standard enzyme-linked immunosorbent assay (ELISA) kits, respectively.

### HE Staining of Renal Tissue

Kidneys were fixed in 4% paraformaldehyde for 24h and then embedded in paraffin. Tissues were then cut into 3μm sections for hematoxylin and eosin (H&E) staining. Glomerular pathology and renal interstitial infiltration were examined under light microscope fields.

### Flow Cytometric Analysis

Spleens and kidneys were harvested while single-cell suspensions were prepared by passing the tissues through a 70-μm cell strainer. For analysis of peritoneal macrophages, peritoneal lavage fluid was obtained after flushing the peritoneal cavity with 4 ml of cold PBS. Cells were blocked with anti-CD16/CD32 Abs and then stained for surface makers, including anti-CD45-BV605, anti-F4/80-APC, anti-CD11b-V450, anti-CD206-PE-cy7, anti-CD86-FITC, Anti-PD-L1-Percyp-efluor and anti-PD-L2-PE Abs. The populations of CD206^+^, CD86^+^, PD-L1^+^ and PD-L2^+^ cells in spleen/peritoneal F4/80^+^CD11b^+^macrophages or *in vitro* treated Raw264.7 cells were also analyzed. The CD86^+^PD-L1^+^ and CD206^+^PD-L2^+^ macrophages in the kidney finally were calculated.

### Immunofluorescence

For immunofluorescence stainings of renal macrophages, kidney tissues were embedded in OTC and cut into 3μm-thick sections. Sections then were fixed in precooled acetone for 10 min and blocked with 0.5% BSA containing 0.1% Tween 20 for 1h. For double-stainings of immunofluorescence, tissues were incubated with mixed primary antibodies (mouse F4/80 with rabbit iNOS, or mouse F4/80 with rabbit Arg1) that were diluted with blocking buffer at 4°C overnight. The next day, the slides were incubated with a mixture of fluorescence- labeled goat anti-rabbit and anti-mouse secondary antibodies for 1h. Pictures were acquired using an inverted fluorescence microscope or confocal laser microscopy (400×).

### Cell Culture

Raw264.7 cell lines were purchased from Cell Resource Center of Shanghai Institute of Life Sciences, Chinese Academy of Sciences (Shanghai, China). Cells were seeded in 12-well or 24-well plates at a concentration of 2×10^5^ or 3×10^5^ per well, respectively. 12 hours after incubation, cells were pretreated with IL-4 (20 ng ml−1) or LPS (50 ng·ml−1) plus IFN-γ (20 ng·ml−1) for 12h to generate M1 and M2 macrophages and maintained for another 48h with or without TGP (20 ug/ml, or 40 ug/ml, dissolved in DMSO), and with or without 100nM STAT6 inhibitor, AS1717499, which was dissolved in DMSO. The final concentration of DMSO in all wells was 0.1%. Cells finally were harvested for whole cell lysates or for flow cytometric analysis.

### Western Blot

Whole protein was obtained using RAPA buffer and protein concentrations were measured using BCA Kit. Samples were run on 10% SDS-PAGE gels and transferred onto a PVDF membrane. After blocking with 5% milk prepared in TBST buffer, membranes were incubated at 4°C overnight with the primary anti-p-STAT6, anti-p-STAT1, anti-PD-L1 and anti-PD-L2 Abs. The next day, membranes were washed and incubated with HRP-labeled secondary antibody for 1 h at room temperature. For detecting of STAT6 and STAT1 and GAPDH, membranes were stripped and reblotted with anti-STAT6, anti-STAT1 and GAPDH Abs. Signals were revealed by ECL system and analyzed using Image J Program software.

### Statistical Analyses

Data were presented as the mean ± SD and comparisons of the means were analyzed by one-way ANOVA using GraphPad Prism 6 (GraphPad Software, La Jolla, CA, USA). A value of P < 0.05 was considered statistically significant.

## Results

### TGP Treatment Improves Renal Function and Pathology in Mice With Pristane-Induced Lupus Nephritis (LN)

**Figure 1:**
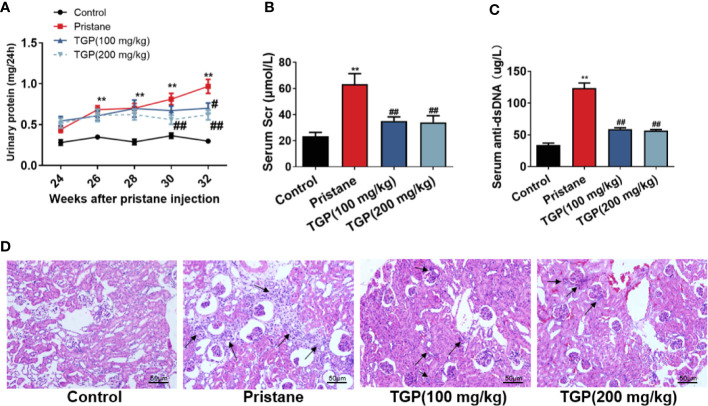
TGP treatment improves renal pathology and function in mice with pristane-induced lupus nephritis. **(A)** 24 weeks after pristane injection, 24-h urinary protein was determined every two weeks and mice with >0.4 mg/24 h urinary protein were also treated with total glucosides of paeony (TGP) in subsequent experiments starting at the same 24-week point. The effects of TGP on kinetics of 24-h urinary protein in pristane-induced lupus mice were observed. In addition, eight weeks after TGP treatment, the serum creatinine (Scr) **(B)** and serum anti-ds-DNA **(C)** were measured using sarcosine oxidase creatinine assay kits and murine anti-dsDNA standard enzyme-linked immunosorbent assay (ELISA) kits, respectively. Data are presented as means ± SD (n=5-6, ** p < 0.01 compared with control, and ^##^p < 0.01 or ^#^p < 0.05 compared with pristane-treated mice). **(D)** HE staining of the kidney in the pristane-induced lupus mice 8 weeks after TGP treatment (200×, with black arrows pointing to infiltrating immune cells).

It’s well known that one-time injection of pristane can induce multiple pathological manifestations resembling human lupus nephritis, including autoantibodies, progressive proteinuria and renal injury. Here, we established a lupus nephritis animal model in C57BL/6 mice *via* intraperitoneal injection of pristane. 24 weeks after injection, almost all mice had an increase in autoantibodies to a high level of around 100 ug/L (data not shown). Almost 80% mice developed mild proteinuria of around 0.45 mg/24h 24 weeks after the injection and progressed to a level of 0.6 mg/24h at the point of 26 weeks with a statistical significance compared with that of normal control mice (**Figure 1A**). However, although mice treated with TGP also showed an increase of proteinuria compared to normal control group. TGP treatment at 200 mg/kg significantly decreased the pristine-induced proteinuria 6 or 8 weeks after TGP treatment (30 and 32 weeks after pristane injection, respectively), while TGP treatment at 100 mg/kg reduced the proteinuria only 8 weeks after TGP treatment (32 weeks after pristane injection) (**Figure 1A**). Meanwhile, TGP treatment for 8 weeks also decreased the serum levels of creatinine (Scr, **Figure 1B**) and anti-dsDNA Ab (**Figure 1C**). Moreover, we performed HE staining of renal tissue and found that mice injected with pristane exhibited obvious interstitial and glomerular cellular infiltration with proliferative glomerular basilar membrane and mesangium (**Figure 1D**). TGP treatment at both 100 mg/kg and 200 mg/kg significantly attenuated the cellular infiltration and improved glomerular injury induced by the pristane.

### TGP Promotes Macrophage M2 Polarization in Mice With Pristane-Induced LN

**Figure 2:**
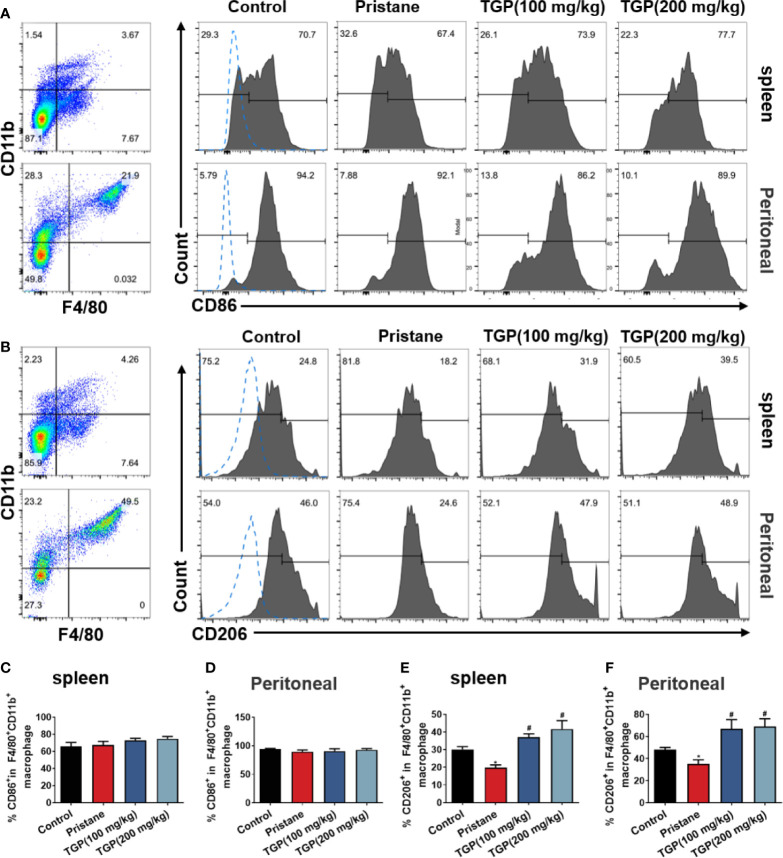
TGP treatment promotes macrophage M2 polarization in pristane-induced lupus mice. Single-cell suspensions from spleens and peritoneal lavage fluids were collected 8 weeks after TGP treatment following pristane injection 24 weeks ago. The effects of TGP on the frequency of CD86^+^ **(A)** and CD206^+^ **(B)** subsets in the splenic and peritoneal F4/80^+^CD11b^+^ macrophages were analyzed *via* flow cytometry. **(C, D)** The percentages of CD86^+^ subset in splenic and peritoneal F4/80^+^CD11b^+^ macrophages are shown as means ± SD. **(E, F)** Shown also are the means ± SD of the percentages of CD206^+^ subset in splenic and peritoneal F4/80^+^CD11b^+^ macrophages. (n=6, ^*^p < 0.05 compared with control, and ^#^p<0.05 compared with pristane-treated mice).

In general, macrophages are divided into the “classically” activated macrophages (M1) and “alternatively” activated macrophages (M2). An important approach to inhibiting the immune responsiveness in autoimmune diseases includes an induction of M2 macrophage polarization. To determine whether TGP affects macrophage polarization in LN mice, M1 and M2 macrophages in spleen and peritoneal lavage fluid were detected *via* FACS analysis. As shown in **Figure 2**, pristane induced a marked decrease in the subset of F4/80^+^CD11b^+^CD206^+^ M2 but not F4/80^+^CD11b^+^CD86^+^ M1 macrophages. While TGP did not alter the frequency of F4/80^+^CD11b^+^CD86^+^ M1 macrophages in spleen and peritoneal lavage fluid of LN mice (**Figures 2A, C, D**), it (either 100 or 200 mg/kg) significantly increased the percentage of F4/80^+^CD11b^+^CD206^+^ cells in both spleen and peritoneal lavage fluid compared to that of pristane alone group (**Figures 2B, E, F**). These data suggest that TGP promotes macrophage M2 polarization.

### TGP Upregulates Expression of PD-Ls on Macrophages in Pristane-Induced LN Mice

**Figure 3:**
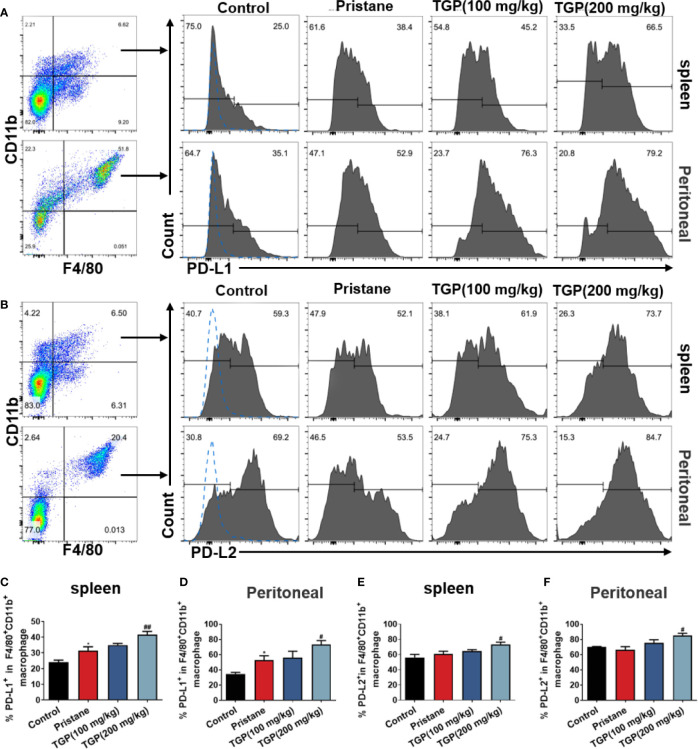
TGP treatment upregulates macrophage expression of PD-Ls in pristane-induced lupus mice. Single-cell suspensions from spleens and peritoneal lavage fluids were collected 8 weeks after TGP treatment following pristane injection 24 weeks ago. The effects of TGP on the frequency of PD-L1^+^ **(A)** or PD-L2^+^ **(B)** subsets in splenic and peritoneal F4/80^+^CD11b^+^ macrophages were determined *via* FACS. **(C, D)** The percentages of PD-L1^+^ subset in splenic and peritoneal F4/80^+^CD11b^+^ macrophages are shown as means ± SD. **(E, F)** Shown are the means ± SD of the percentages of PD-L2^+^ subset in splenic and peritoneal F4/80^+^CD11b^+^ macrophages. (n=5-6, *p < 0.05 compared with control, and ^##^p < 0.01 or ^#^p < 0.05 compared with pristane treated mice).

The role of PD1/PD-L1/PD-L2 pathway in regulating immunological tolerance and immune-mediated tissue damage has been highlighted previously. PD-L1 and PD-L2 are expressed on macrophages, monocytes, endothelial cells and cancer cells, and they influence both macrophage polarization and macrophage function. Here, we asked whether TGP would affect the PD-Ls expression on macrophages. As displayed in **Figure 3**, pristane alone upregulated PD-L1, but not PD-L2, expression on F4/80^+^CD11b^+^ macrophages in spleen and peritoneal lavage fluid of LN mice. TGP treatment at high, but not low, doses significantly increased the frequency of F4/80^+^CD11b^+^PD-L1^+^ and F4/80^+^CD11b^+^PD-L2^+^ macrophages in both spleen and peritoneal lavage fluid. These data suggest that an increase in PD-L1/PD-L2 expression on macrophages may contribute to one of the mechanisms responsible for the effects of TGP on lupus nephritis.

### TGP Promotes Renal PD-Ls^+^ Macrophage Infiltration in Pristane-Induced LN Mice

**Figure 4:**
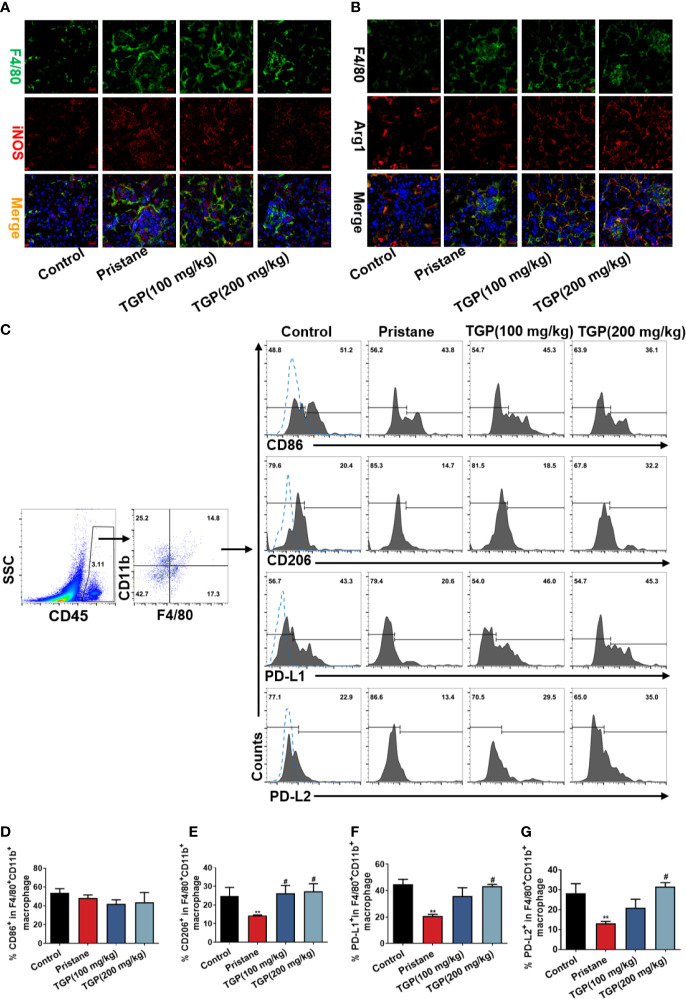
TGP treatment increases renal PD-Ls^+^ macrophage infiltration in pristane-induced lupus mice. **(A)** Immunofluorescence double-stainings for F4/80 and iNOS or **(B)** for F4/80 and Arg1 in the kidney of pristane-induced LN mice 8 weeks after TGP treatment (400×). **(C)** Kidney infiltrating cells were isolated 8 weeks after TGP treatment following pristane injection 24 weeks ago and the total infiltrating macrophages were gated on CD45^+^F4/80^+^CD11b^+^ population. The percentages of kidney infiltrating CD86^+^ **(D)**, CD206^+^ **(E)**, PD-L1^+^ **(F)** or PD-L2^+^ **(G)** macrophages were determined *via* flow cytometer, as shown by the bar graphs, in which data are presented as means ± SD (n=5-6, ^**^p < 0.01 compared with control, and ^#^p < 0.05 compared with pristane-treated mice).

Macrophages are a predominant population of immune cells that infiltrate the kidney during lupus nephritis. Thus, infiltration of macrophages into the kidney was analyzed in pristane-induced LN mice. Immunofluorescence staining showed that there were very few F4/80^+^ macrophages in normal control mice. In contrast, extensive F4/80^+^ macrophage infiltration was seen in the tubular and glomeruli of pristane-treated mice, indicating the likely participation of macrophages in the renal pathology of lupus nephritis. TGP treatment did not appear to alter F4/80^+^ macrophage infiltration compared with the pristane group. However, as shown in **Figure 4**, TGP treatment at doses of 200 mg/kg decreased the F4/80^+^iNOS^+^ macrophages (**Figure 4A**), but increased F4/80^+^Arg1^+^ macrophage infiltration in the kidney (**Figure 4B**), when compared to the pristane-alone group. Further, the percentages of CD86^+^/CD206^+^ and PD-L1^+^/PD-L2^+^ macrophages in the kidney were evaluated by flow cytometry. As shown in **Figures 4C–G**, among the total F4/80^+^CD11b^+^ macrophages, CD206^+^, PD-L1^+^ and PD-L2^+^ macrophage subsets were decreased in pristane-treated group, compared with normal control group, while TGP treatment significantly increased the percentages of CD206^+^, PD-L1^+^ and PD-L2^+^ macrophages in the kidney compared to pristane-alone group. However, neither pristane nor TGP treatment had a significant effect on the frequency of CD86^+^ macrophage subset. Therefore, TGP preferentially promoted infiltration of macrophages expressing PD-L1/PD-L2 in the kidney of pristane-treated LN mice.

### TGP Enhances M2 Polarization of PD-Ls^+^ Macrophages *In Vitro*

**Figure 5:**
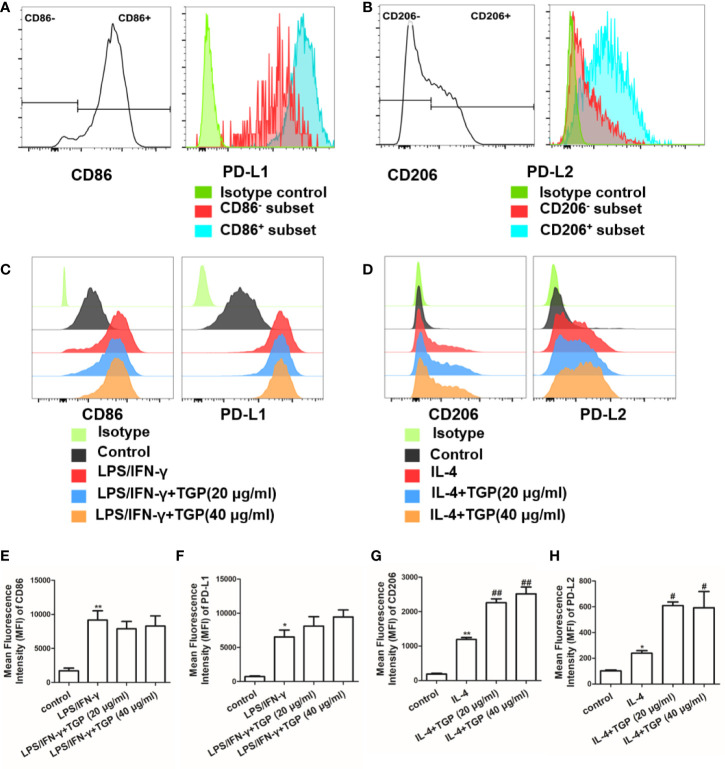
TGP promotes PD-Ls-expressing macrophage polarization *in vitro*. **(A)** Representative FACS plots show the expression of PD-L1 in CD86^-^ and CD86^+^ Raw264.7 macrophages stimulated with LPS/IFN-γ. **(B)** Representative FACS plots display the expression of PD-L2 in CD206^-^ and CD206^+^ Raw264.7 macrophages stimulated with IL-4. **(C)** The expression of CD86 or PD-L1 in LPS/IFN-γ-treated Raw264.7 cells was determined *via* FACS. **(D)** The expression of CD206 or PD-L2 in IL-4-treated Raw264.7 cells was determined *via* FACS. **(E–H)** Data are shown as the mean percentages of CD86 **(E)** and PD-L1 **(F)** expression in LPS/IFN-γ-treated Raw264.7 cells or CD206 **(G)** and PD-L2 **(H)** expression in IL-4-treated Raw264.7 cells. Data are presented as means ± SD (n=3-4, ^**^p<0.01 or ^*^p < 0.05 compared with control, and ^##^p < 0.01 or ^#^p < 0.05 compared with LPS/IF-γ or IL-4 group without TGP).

We then determined the effects of TGP on macrophage polarization *in vitro*. Raw264.7 cells were pretreated with LPS/INF-γ or IL-4 to mimic the *in vivo* polarization. Expression of PD-L1/PD-L2 was determined *via* flow cytometry and represented as mean fluorescence intensity (MFI). As shown in **Figures 5A, B**, at basal state, expression of PD-L2 on Raw264.7 cells was significantly lower than that of PD-L1. Although LPS/INF-γ treatment increased expression of CD86 and PD-L1 on macrophages, addition of TGP did not further alter their expression compared to LPS/INF-γ group (**Figures 5C, E, F**). However, macrophages alternatively activated by IL-4 exhibited an increase in PD-L2, while addition of TGP further augmented their expression of both CD206 and PD-L2 compared with IL-4 alone group (**Figures 5D, G, H**). Thus, TGP significantly increases CD206 and PD-L2 expressions in IL-4 treated macrophages but not CD86 and PD-L1 expressions in LPS/INF-γ-treated ones.

### TGP Increases PD-L2 Expression on Macrophages *via* Phosphorylating STAT6

**Figure 6:**
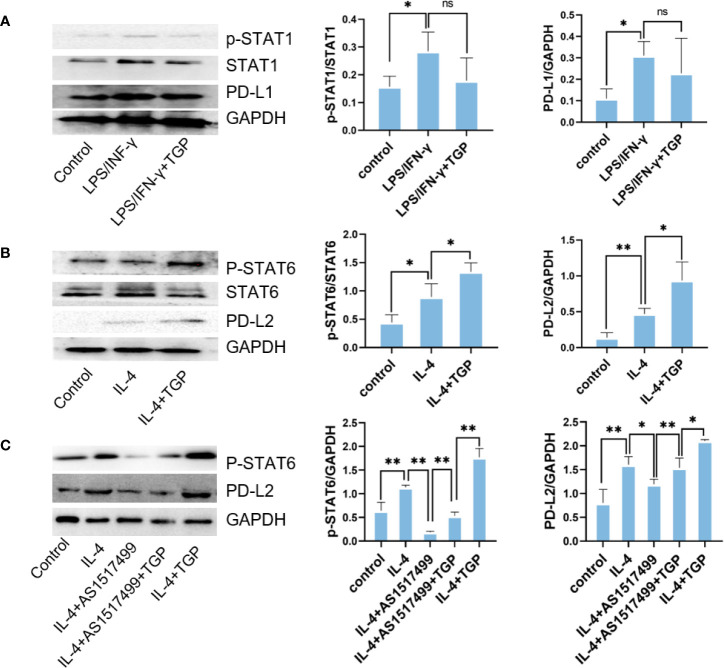
TGP augments PD-L2 expression on macrophages *via* phosphorylating STAT6. **(A)** Raw264.7 cells were pretreated with LPS/IFN-γ for 12 h and treated with TGP for another 48 h, then protein expressions of p-STAT1, STAT1 and PD-L1 were detected *via* western blot analyses. **(B)** Raw264.7 cells were pretreated with IL-4 for 12 h and treated with TGP for another 48 h, the protein expressions of p-STAT6, STAT6 and PD-L2 were then detected *via* western blot analyses. **(C)** To determine if TGP regulates PD-L2 expression *via* STAT6 pathway, Raw264.7 cells were treated with a STAT6 inhibitor, AS1517499, and detected for the expressions of p-STAT6 and PD-L2. Overall, GAPDH was used as internal references. The expressions of p-STAT1, STAT1, PD-L1, p-STAT6, STAT6 and PD-L2 were calculated relatively to GAPDH. Data are presented as means ± SD (n=3, ^**^p<0.01 or ^*^p<0.05, as indicated for direct comparisons, ns, not significant).

It has been reported that STAT6 is involved in IL-4-mediated macrophage M2 polarization and PD-L2 expression, while STAT1 is involved in LPS/IFN-γ mediated macrophage M1 polarization and PD-L1 expression. Therefore, we adopted an *in vitro* model of macrophage polarization to see how the polarization of macrophages alters PD-Ls expression and whether TGP induces PD-L2 expression on macrophages by activating STAT6 signaling pathway. As shown in **Figure 6**, LPS/IFN-γ induced phosphorylation of STAT1 and upregulated PD-L1 expression. TGP only slightly reduced p-STAT1 and PD-L1 expression but with no statistical significance (**Figure 6A**). IL-4 upregulated both p-STAT6 and PD-L2 expression in macrophages, while TGP treatment further enhanced IL-4-induced phosphorylation of STAT6 and PD-L2 expression compared to IL-4 alone group (**Figure 6B**). More importantly, the induction of PD-L2 expression by TGP was abolished by a STAT6 inhibitor, AS1517499 (**Figure 6C**), indicating that TGP exerts its effects on macrophage polarization and PD-L2 expression mainly through activating STAT6/PD-L2 signaling pathway.

## Discussion

Renal macrophage infiltration represents a prominent feature of inflammation and immunopathology for lupus nephritis. Macrophage phenotypes and functions directly impact the progression and prognosis of lupus nephritis. This present study demonstrated that few PD-L1^+^ and PD-L2^+^ macrophages infiltrated in the kidney of mice with pristane-induced lupus nephritis. However, TGP treatment induced both PD-L1^+^ and PD-L2^+^ macrophages with less renal infiltration of iNOS^+^ macrophages, thereby ameliorating lupus nephritis in pristane-treated mice. Furthermore, using an *in vitro* macrophage polarization model, our study revealed that STAT6 signaling contributed to the expression of PD-L2 on macrophages. These results suggest that induction of PD-Ls^+^ macrophages represents a strategy for treating lupus nephritis or even other autoimmune kidney diseases.

TGP is a group of glycosides extracted from the dried root of *Paeonia lactiflora Pallas*, and it has been approved for marketing in China to treat rheumatoid arthritis. TGP have been widely used in clinic for the treatment of inflammatory diseases and autoimmune diseases, including rheumatoid arthritis, SLE, psoriasis and Sjögren’s syndrome (21, 22). Moreover, previous studies have shown that TGP treatment attenuates renal inflammation in experimental diabetes (23, 24). In this study, we demonstrated that TGP attenuated proteinuria, decreased serum Scr and anti-dsDNA, and improved renal histology in mice with pristane-induced lupus nephritis. Therefore, TGP treatment could be beneficial to patients with lupus nephritis. Researchers have also shown that TGP was effective in modulating activation of various immune cells, including Treg, Th17, B cells, DCs and macrophages. It was reported that TGP treatment inhibited macrophage proliferation and accumulation in the kidney of diabetic rats (24). In our present study, TGP treatment promoted M2 polarization to CD206^+^PD-L2^+^ macrophages *via* STAT6 signaling. These results indicate that TGP treatment not only affects the macrophage proliferation, but also alters the polarization of macrophages and their phenotypes.

Macrophages are a main subset of immune cells infiltrating the kidney in lupus nephritis, dominating the renal inflammation and immune responses. Here, using pristane-induced lupus mouse models, we found large infiltrates of F4/80^+^ macrophages in both renal interstitium and glomeruli, which was consistent with the results from renal histology of patients (25). The functions of macrophages rely on their phenotypes, which are classically defined as M1 and M2 macrophages. Compelling evidence indicates that the M1 macrophage infiltration is strongly correlated with the severity of renal injury in both acute (26) and chronic kidney disease (27, 28). However, M2 macrophages were predominant during the anti-inflammatory or repair process. In the present study, mice treated with pristane exhibited a remarkable decrease in M2 macrophages in the spleen, peritoneal lavage fluid and kidney. In contrast, the M1 macrophage infiltration was increased, but only in the kidney. This discordance between renal tissues and lymphoid organs might be due to the difference in local immune microenvironment and sources of macrophages. TGP treatment for 8 weeks significantly increased the CD206^+^ frequency in both spleen and peritoneal lavage fluid. In addition, TGP enhanced the kidney infiltration of F4/80^+^Arg1^+^ macrophages and decreased that of F4/80^+^iNOS^+^ macrophages. Our data suggest that TGP treatment can rebalance the M1/M2 polarization in the kidney of the mice with lupus nephritis.

Besides polarization, the phenotype changes during polarization play an important role in the macrophage function. The programmed death-1 (PD-1) and its ligands (PD-Ls: PD‐L1 and PD-L2) are acknowledged as critical immunosuppressive factors in immune responsiveness and autoimmunity (29). Deficiency of PD-Ls is strongly associated with the pathogenesis of many autoimmune diseases, including lupus nephritis, Type 1 diabetes mellitus, RA and so on (29–31). PD-L1 is constitutively and widely expressed on T cells, B cells, macrophages, monocytes, and some nonhemopoietic parenchymal cells. In contrast, PD-L2 expression is limited to activated M2 macrophages and DCs (32). More importantly, at the same ligand concentration, PD-L1 had a delayed interaction with its receptor PD-1 compared with PD-L2 (33). In this study, we demonstrated that PD-L1 was mainly induced by LPS/IFN-γ and expressed on CD86^+^ macrophages, while PD-L2 was mainly induced by IL-4 and expressed on CD206^+^ macrophages. Previous studies have shown that the expressions of PD-Ls on monocyte/macrophage contribute to the induction of Tregs and suppression of effector T cells (9, 10). In addition, previous research has also indicated potential associations between PD-Ls expression and polarization, implying that macrophages highly expressing PD-Ls are more prone to M2 polarization (34–36). Taken together, these data suggest that PD-Ls influence both the polarization and immunomodulatory function of macrophages. In this study, we showed that pristane increased PD-L1 expression in splenic and peritoneal macrophages with no effect on PD-L2 expression. However, we found a different expression pattern of PD-Ls in the renal macrophages. Both PD-L1 and PD-L2 expressions in kidney-infiltrating macrophages were actually decreased in pristane-treated mice compared with normal control mice. It was previously reported that in mice with nephrotoxic serum nephritis, PD-L1 and PD-L2 were largely responsible for the protective role of macrophages in attenuating the kidney disease, although an increase in PD-L1 and PD-L2 expressions on renal macrophages was seen (37). We speculate that the differential expression pattern of PD-Ls between nephrotoxic serum nephritis and pristane-induced lupus nephritis may be due to the different timing for establishing models. But, in both models, TGP treatment significantly increased both PD-L1 and PD-L2 expressions in splenic, peritoneal and renal infiltrating macrophages, confirming the actively immunomodulatory roles of PD-Ls on macrophages in inflammatory kidney diseases.

We also studied the effects of TGP on macrophage polarization and the molecular mechanisms underlying its effects on PD-Ls expression. It has been reported the PD-L1 is mainly regulated by Th1 cytokines and STAT1 pathway (38, 39), while PD-L2 is modulated largely by Th2 cytokines and STAT6 pathway (11, 40). In present study, we found that TGP treatment activated the IL-4/STAT6/PD-L2 pathway in M2 macrophages. Unlike its effects on PD-L1 in pristane-induced mice *in vivo*, TGP treatment had no significant effects on STAT1 signaling or PD-L1 expression *in vitro*. The reason for this discrepancy remains to be determined. But it still suggests that TGP does not directly impact STAT1 pathway or PD-L1 expression on M1 macrophages *in vitro*. TGP could alter their PD-L1 expression *in vivo via* indirect effects on other immune cells, which in turn interact with the macrophages.

In conclusion, we demonstrated that TGP treatment not only promoted the macrophage M2 polarization but also increased the PD-L1 and PD-L2 expressions on macrophages, thereby ameliorating the renal inflammation and injury in lupus nephritis. These findings will enhance our understanding of the immunomodulatory effects of TGP on autoimmune diseases. Further in-depth studies on how and why TGP modulates PD-L1 and PD-L2 expressions on the macrophages are warranted, and may help improve the treatment of various autoimmune diseases in the near future.

## Data Availability Statement

The original contributions presented in the study are included in the article/supplementary material. Further inquiries can be directed to the corresponding authors.

## Ethics Statement

The animal study was reviewed and approved by The Animal Ethics Committee of Guangdong Provincial Academy of Chinese Medical Sciences.

## Author Contributions

C-LL performed experiments and wrote the primary draft. HJ, WF, and HL performed some experiments. LH analyzed some data and edited the manuscript. YC, QZ, and FZ analyzed some data. C-JL provided general ideas and crucial reagents. ZD oversaw the study and edited the manuscript. All authors contributed to the article and approved the submitted version.

## Funding

This work was supported by National Natural Science Foundation of China (81803821), Natural Science Foundation of Guangdong Province (2017A030310127, 2018A030313256, 2018A030310530 and 2019A1515110741), the Specific Research Fund for TCM Science and Technology of Guangdong Provincial Hospital of Chinese Medicine (YN2019MJ03, YN2019QJ07 and YN2019QJ02) and the Specific Fund of State Key Laboratory of Dampness Syndrome of Chinese Medicine (SZ2020ZZ28).

## Conflict of Interest

The authors declare that the research was conducted in the absence of any commercial or financial relationships that could be construed as a potential conflict of interest.
